# Prevention of atelectasis secondary to propofol-based general anesthesia by application of continuous positive airway pressure to children with neuroblastoma undergoing computerized tomography: a quality improvement project

**DOI:** 10.1186/s40981-019-0297-z

**Published:** 2019-11-25

**Authors:** Rebecca D. Margolis, Sean J. Gamble, Jimmy J. Hoang, Makoto Nagoshi

**Affiliations:** 1 Department of Anesthesiology Critical Care Medicine, Children’s Hospital Los Angeles, Keck School of Medicine, University of Southern California, 4650 Sunset Boulevard, Los Angeles, CA 90027 USA; 2 Present address: Department of Anesthesiology, Phoenix Children’s Hospital, 645 E Missouri Ave, Suite 300, Phoenix, AR 85012 USA

Atelectasis is a well-known complication of general anesthesia (GA) and intravenous propofol sedation [1–4]. Incidence of anesthesia-induced atelectasis is higher in younger age [4, 5]. In children with neuroblastoma, false uptake of metaiodobenzylguanidine (MIBG) in the lung due to atelectasis has been reported [6], which can obscure images of pulmonary metastases [7]. However, the incidence and severity of atelectasis in children receiving SPECT/PET/CT has not been delineated. Due to concerns of atelectasis confounding imaging quality in younger children, we implemented a new protocol to reduce atelectasis risk with continuous positive airway pressure (CPAP).

## Methods

**Fig. 1:**
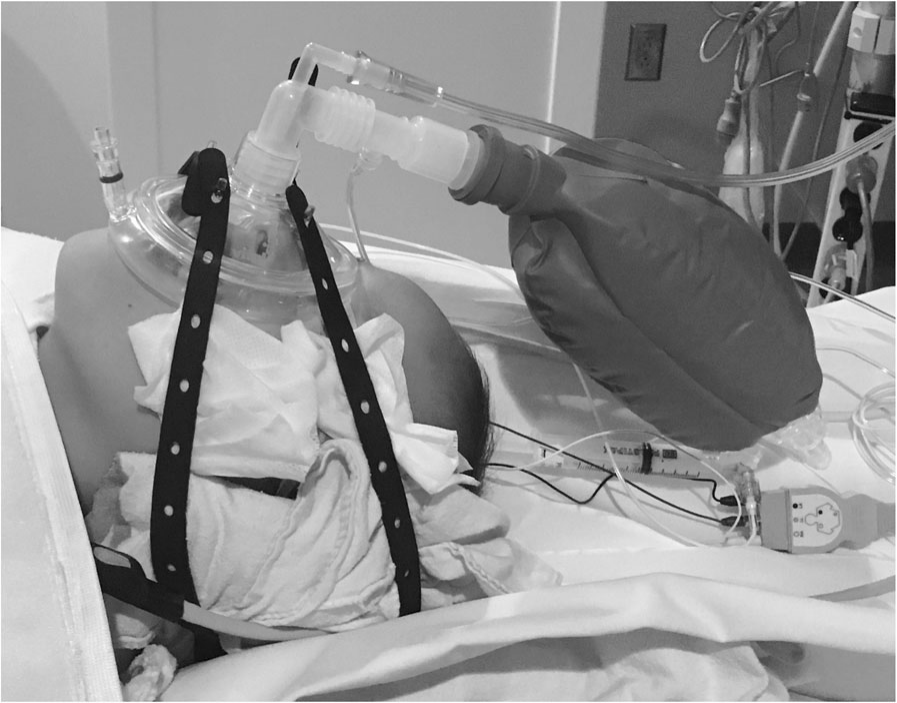
A child with anesthesia mask and head strap connected to Jackson-Rees bag maintaining CPAP

After IRB approval, imaging of children with neuroblastoma from the ages of 1 month to 10 years old was analyzed. Prior to the protocol change, we used an intravenous propofol infusion (150–250 mcg/kg/min) with nasal cannula. Following the protocol change, children’s airway was supported with a laryngeal mask airway (LMA) or an anesthesia mask with a strap connected to a Jackson-Rees circuit (Fig. 1). Air blender (FiO_2_ < 30%) was used to prevent absorption atelectasis [8]. Higher propofol infusion rate (250–300 mcg/kg/min) was used to allow children to tolerate in situ airway. Air flow and AP valve in the circuit were adjusted to maintain CPAP (5 to 10 cmH_2_O) [5]. We titrated infusion rate according to respiratory parameter (SpO2 > 95%) and hemodynamical parameter (mean arterial pressure above 40 mmHg).

A single, blinded pediatric radiologist graded the severity of atelectasis according to previously described scale [7] and determined whether there was significant atelectasis to confound image quality or not.

Statistics were calculated using Mcnemar’s test due to paired observations from children receiving imaging both pre- and post-protocol implementation.

## Results

Nine children underwent both pre- and post-protocol, but demographics of those at different time points of SPECT/PET/CT were very similar. The incidence of significant atelectasis in the “pre-protocol” sedation was 3/9 (33.3%) vs 0/9 patients (0.0%) in the “post-protocol” (*p* value = 0.04).

## Discussion

Our observations among the nine children who underwent intravenous propofol infusion with and without CPAP at different time points showed a significant reduction in the incidence and severity of atelectasis with CPAP. Our new protocol is unique because we were able to achieve CPAP without the use of an anesthesia ventilator. The results of our study need to be considered in light of its limitations. Due to a small sample size and the retrospective nature of the study, we could not pin-point the role of patient factors. The grading scale was developed by a single radiologist and has not been validated. We encourage larger, randomized studies to elucidate the effect of propofol anesthesia on the imaging quality in the abovementioned patient population, as well as other cost-effective, reproducible methods to reduce the severity of atelectasis in this patient population.

In conclusions, adding CPAP to an intravenous propofol infusion using a Jackson-Rees bag and an air blender can decrease the incidence and severity of atelectasis in children and can be accomplished without an anesthesia machine.

## Data Availability

They are available as electric files upon reasonable request.

## Abbreviations

GA: General anesthesia
CPAP: Continuous positive airway pressure
CT: Computerized tomography
LMA: Laryngeal mask airway
MIBG: Metaiodobenzylguanidine
NC: Nasal cannula
PET: Positron emission tomography
SPECT: Single-photon emission computed tomography
